# Omphalomesenteric Duct Cyst in an Omphalocele: A Rare Association

**DOI:** 10.12669/pjms.293.3581

**Published:** 2013

**Authors:** Yousuf Aziz Khan, Mumtaz Ahmed Qureshi, Jamshed Akhtar

**Affiliations:** 1 Yousuf Aziz Khan, MBBS, FCPS (Paediatric Surgeon), Department of Paediatric Surgery, National Institute of Child Health, Rafiquee Shaheed Road, Karachi – 75510, Sind, Pakistan.; 2 Mumtaz Ahmed Qureshi, MBBS, FCPS (Paediatric Surgeon), Department of Paediatric Surgery, National Institute of Child Health, Rafiquee Shaheed Road, Karachi – 75510, Sind, Pakistan.; 3 Jamshed Akhtar, MBBS, FCPS (Paediatric. Surgeon). Department of Paediatric Surgery, National Institute of Child Health, Rafiquee Shaheed Road, Karachi – 75510, Sind, Pakistan.

**Keywords:** Omphalomesenteric duct remnants, Omphalocele, Omphalomesenteric duct cyst, Newborn

## Abstract

Omphalomesenteric duct (OMD) remnants and omphalocele are not infrequently seen in paediatric patients. In most of the cases, OMD remnant in an omphalocele is a Meckel’s diverticulum; however rarely there may be other lesions. A one-day old male baby underwent surgery for omphalocele. At exploration a 10 x 12 cm cyst containing gut contents was found as the content of the omphalocele, with proximal and distal ileal loops running in continuity with it. Resection of the cyst with end to end primary gut anastomosis was done. Baby also had complex associated cardiac anomalies and died few days after surgery due to sepsis.

## INTRODUCTION

**Omphalomesenteric duct is a normal embryological structure that connects the fetal midgut with the yolk sac. It usually regresses by 9** ^th^ **week of gestation. However if that does not happen, a spectrum of anomalies may result.** ^1^ **Remnants of OMD may be present in 2% of the population with Meckel’s diverticulum (MD) as the commonest of remnants. OMD remnants may form part of an omphalocele however, omphalocele containing an OMD cyst is extremely rare and very few cases have been reported.** ^2^^,^^3^ **Some of these cysts are serous, probably occurring due to the sequestration of the OMD remnants during intrauterine life. Meconium or faecal cysts probably develop as a consequence of partial obstruction at the neck of the umbilical defect where gut contents can flow into the OMD and forms a large cyst. Herein we report a case of a newborn with an omphalocele in whom an omphalomesenteric cyst was found.**

## CASE REPORT

**Fig.1:**
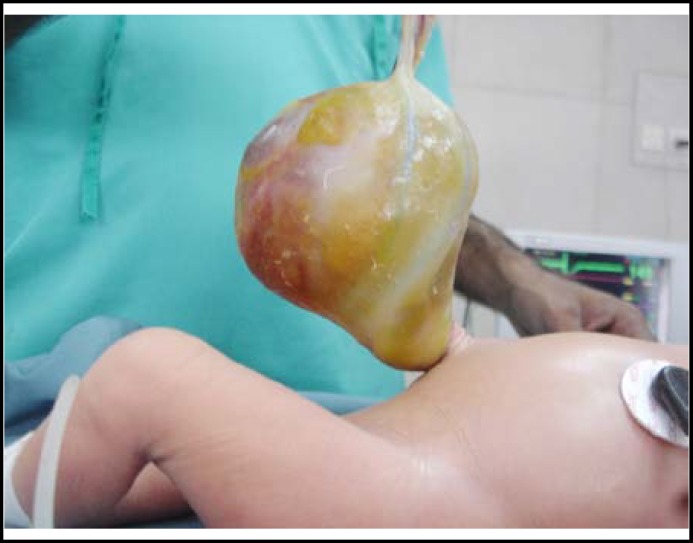
Large omphalocele containing OMD cyst

**A one-day old, full term male, weighing 2.6 kg, was referred with a swelling at umbilical region. Baby was delivered at a local hospital through spontaneous vaginal delivery. On arrival, he had normal morphological features. There was a large omphalocele about 10 x 12 cm, covered with a thin, intact, semi-transparent membrane with a narrow pedicle of around 3 cm diameter (** Fig.1 **). Umbilical clamp was well away from the omphalocele. The small bowel loops and a thick sac like structure were visible through the thin membrane. Rest of the neonatal examination was unremarkable.**

**Fig.2:**
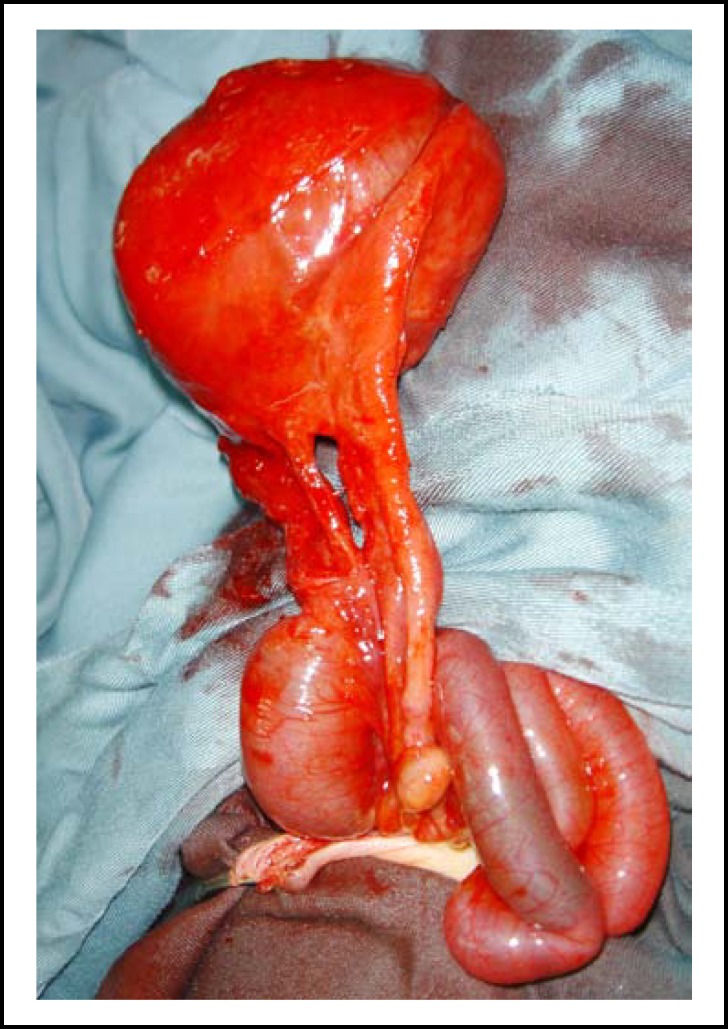
Per operative picture showing cystically dilated segment of bowel in the region of Meckel’s diverticulum, in continuity with proximal and distal ileal loops

**Echocardiography showed a large ventricular septal defect with overriding of aorta, hypertrophied right ventricle, infundibular pulmonary stenosis and bunching of atrial septum – correlated with Tetralogy of Fallot. After optimization of the general condition surgery was done. At surgery, a 10 x 12 cm meconium filled cyst located at the distal ileum was found as the major content of the sac, along with small intestinal loops. The proximal and distal ileal loops were communicating with the cyst. (** Fig. 2 **and** 3 **) Rest of the bowel had no gross anomaly. Excision of cyst with primary end-to-end ileo-ileal anastomosis was performed.**

**Post operative course was stormy. During his stay at PICU the baby developed uncontrolled septicaemia and died in spite of best of efforts. Histopathology of the excised specimen revealed wall of small intestine with thin walled blood vessels surrounded by fibromyxomatous tissue. Ectopic mucosa was not identified.**

## DISCUSSION

**Omphalomesenteric duct cyst is infrequently reported.** ^2^^,^^4^ **Vane et al found only three cases of OMD cyst in 217 children with OMD remnants.** ^5^ **It may be found attached to the umbilicus, bowel or both.**^4^** Males have higher incidence than females (4:1) as with other OM anomalies, and are not associated to maternal age, gravidity, race or prematurity.**^4^^,^^6^** It has varied presentations.**^4^^,^^7^^-^^9^ **In newborns, OMD remnants may present in an omphalocele as reported by Chattopadhyay A et al and Nicol JW et al.**^3^^,^^10^** Ratan SK et al reported a case similar to ours in a newborn but had a cyst containing translucent fluid rather than meconium.**^2^

**Fig.3:**
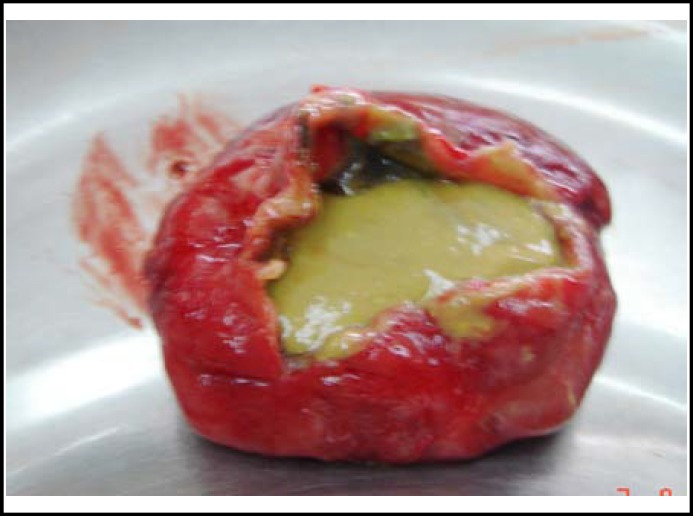
The excised specimen of the cyst containing gut contents

**In the present case, a large cyst was seen in the omphalocele sac, outside peritoneal cavity. It was a localized dilatation in the area of Meckel’s diverticulum and was in communication at both the ends with the small intestine and contained meconium. The possibility of a variant of cystic duplication of the small bowel was ruled out as it was located on anti-mesenteric side of the bowel with no aberrant vascular supply and sharing of the common wall. So an impression of a variant of OMD remnant and possibly omphalomesenteric cyst was made.**

**OMD cysts have a variable diameter and appear as a cystically dilated segment of small bowel. They are usually lined by columnar epithelium resembling that of the small bowel, colon or stomach. Ectopic pancreatic tissue may be present.** ^6^ **The gross and histological characteristics of the specimen in our case were similar to that reported in the literature supporting the possibility of omphalomesenteric cyst. However, no ectopic tissue was identified in our case. In conclusion OMD cyst may be present in a large omphalocele and the prognosis depends upon the associated anomalies.**
